# The Effects of FTO on the Proliferation and Differentiation of Rabbit Preadipocytes

**DOI:** 10.3390/ani15131909

**Published:** 2025-06-28

**Authors:** Jiaxue Wang, Wenqiang Sun, Xianbo Jia, Jie Wang, Hengwei Yu, Songjia Lai

**Affiliations:** College of Animal Science and Technology, Sichuan Agricultural University, Chengdu 611130, China; 13001807553@163.com (J.W.); wqsun2021@163.com (W.S.); jaxb369@sicau.edu.cn (X.J.); wjie68@163.com (J.W.); 18792427097@163.com (H.Y.)

**Keywords:** meat rabbit, FTO, preadipocyte, cell proliferation, differentiation

## Abstract

**Simple Summary:**

With the improvement of living standards and growing health awareness among consumers, rabbit meat has been increasingly recognized as a healthy source of protein, owing to its nutritional profile characterized by high protein, minimal fat content, and negligible cholesterol levels. Compared to other meats, rabbit meat contains higher proportions of unsaturated fatty acids, which are beneficial for human health by helping reduce blood cholesterol and triglyceride levels, thereby lowering the risk of cardiovascular diseases such as coronary heart disease and hypertension. Since the deposition of adipocytes in rabbits directly affects meat quality attributes including tenderness, flavor, and juiciness, this study holds significant importance for improving livestock meat quality and promoting healthier human dietary choices.

**Abstract:**

FTO expression correlates with adipose tissue development in rabbits, yet its regulatory role in preadipocyte differentiation remains poorly understood. Therefore, we employed transcriptome sequencing to identify 422 differentially expressed genes (DEGs) between the FTO overexpression group and the FTO-negative control group. Multi-omics evidence from proliferation assays (CCK-8), protein verification (WB), gene quantification (qPCR), and lipid staining (Oil Red O) established FTO as a positive regulator of rabbit preadipocyte development with gain-of-function enhancing and loss-of-function impeding differentiation. In conclusion, FTO regulates the proliferation and differentiation of rabbit preadipocytes, providing deeper insights into livestock energy metabolism and physiological functions. These findings hold significant implications for healthy animal farming, high-quality livestock product production, and the prevention and control of metabolic diseases in animals.

## 1. Introduction

China, as the world’s leading producer of rabbit meat, dominates global production in both breeding stock and slaughter volumes, with rabbit products enjoying particular popularity in the Sichuan and Chongqing regions. Nutritionally, rabbit meat represents an exceptional source of high-quality protein (20–24% protein content) [1], exhibiting superior digestibility (>85%) compared to other common meats. The unique lipid profile of rabbit meat, characterized by low fat content and high levels of beneficial unsaturated fatty acids (e.g., oleic acid and linoleic acid), contributes to cardiovascular health by reducing serum cholesterol, decreasing triglyceride levels, and mitigating atherosclerosis risk [2]. Furthermore, rabbit meat is rich in essential vitamins and minerals, making it an ideal dietary choice for health-conscious consumers [3]. Cutting-edge research has revealed that m^6^A methylation dynamics, mediated by methyltransferases and demethylases, play a pivotal role in regulating adipocyte differentiation and triglyceride accumulation, thereby influencing fat deposition patterns—a finding that opens new avenues for improving meat quality through epigenetic approaches.

In recent years, RNA modifications have emerged as a major research focus, with N6-methyladenosine (m^6^A) being the most prevalent internal modification in both mRNA and long non-coding RNAs (lncRNAs), characterized by methylation at the N6 position of adenosine residues [4]. This dynamic and reversible modification process is precisely regulated by a sophisticated system comprising methyltransferases (“writers”), demethylases (“erasers”), and m^6^A-binding proteins (“readers”) that collectively maintain the homeostasis of m^6^A levels within cells [5,6,7,8,9].

Emerging evidence has established the demethylase FTO as a critical regulator of adipogenesis. Mechanistically, FTO promotes fat formation by modulating multiple cellular processes including mitotic clonal expansion (MCE) phase progression, metabolite profiles, autophagy, phenotypic transitions, and apoptosis [10,11]. Studies demonstrate that FTO deficiency suppresses adipocyte differentiation during early MCE stages through m^6^A-dependent inhibition of JAK2 expression in porcine and murine preadipocytes [12]. Notably, FTO-knockout mice exhibit enhanced thermogenic capacity and resistance to high-fat diet (HFD)-induced obesity [13]. Complementary work by Tang et al. [14] revealed that decreased m^6^A RNA methylation in livers of HFD-fed mice correlates with elevated FTO expression, while hepatic FTO overexpression directly promotes triglyceride accumulation. These collective findings underscore FTO’s multifaceted role in lipid metabolism regulation.

Therefore, investigating the influence of FTO on the proliferative capacity and differentiation potential of preadipocytes isolated from meat-producing rabbits will provide fundamental insights into the regulatory mechanisms governing adipose tissue development. This research holds significant potential for optimizing livestock fat deposition and improving meat quality and production performance through nutritional interventions and genetic breeding strategies [15].

## 2. Materials and Methods

### 2.1. Meat Rabbit Sourcing and Adipose Tissue Sampling

A total of 300 healthy newborn (0-day-old) Ira meat rabbits were selected for this study. The rabbits were fed a basal diet twice daily (morning and evening) until the end of the experiment at 70 days of age, with ad libitum access to feed and water. Serum, visceral organs, muscle, and perirenal adipose tissues were collected at 0, 35, and 70 days. All samples were immediately frozen in liquid nitrogen and stored at −80 °C for further analysis. Preadipocytes were isolated from the perirenal adipose tissue of humanely slaughtered newborn meat rabbits. All experimental samples were obtained from the Sichuan Agricultural University Practical Base in Bazhong, Sichuan, China.

### 2.2. Cell Culture and Induced Differentiation

Perirenal adipose tissue from neonatal Ira rabbits was aseptically harvested into 6-well plates preloaded with PBS containing 4% penicillin-streptomycin (Gibco, Carlsbad, CA, USA). After microdissection to remove connective tissues and vasculature, adipose fragments were collagenase-digested (Type I) to isolate primary preadipocytes. To purify the preadipocytes, the digestion mixture was sequentially filtered through 70 μm and 40 μm cell strainers, and the filtrate was centrifuged at 1000× *g* for 5 min with the supernatant discarded. The resulting cell pellet was resuspended in fresh growth medium and centrifuged again (1000× *g*, 5 min); this washing procedure was repeated twice to eliminate residual collagenase and cellular debris. Primary preadipocytes were seeded in T25 flasks with growth medium (10% FBS, Gibco, Carlsbad, CA, USA) and cultured at 37 °C/5% CO_2_, with medium changed every 48 h. Upon reaching 80–90% confluency, cells were detached using 1 mL of 0.25% trypsin-EDTA solution for 2 min and passaged at a 1:3 ratio; passaged cells were cryopreserved for subsequent experiments. When cells attained 70–80% confluency, adipogenic differentiation was induced for 4 days using differentiation medium containing 5% FBS (Gibco, Carlsbad, CA, USA), 1 μmol/L Dex, 0.5 mmol/L IBMX, and 10 μg/mL insulin (all from Solarbio, Beijing, China) to monitor lipid droplet formation. Subsequently, the cells were maintained for an additional 4 days in maintenance medium containing 5% FBS and 10 μg/mL insulin, before finally replacing the medium with standard growth medium to obtain mature adipocytes.

### 2.3. Plasmid Construction and Cell Transfection

The FTO coding sequence was ligated into pcDNA3.1(+) (Beijing Tsingke Biotechnology Co., Ltd., Beijing, China) to yield overexpression construct pcDNA3.1-FTO. Short interfering RNAs (SiRNAs) targeting FTO(Si-FTO) were synthesized directly by Beijing Tsingke Biotechnology Co., Ltd. (Beijing, China). When preadipocytes reached approximately 60% confluence, they were transfected with either the pcDNA3.1 empty vector or the pcDNA3.1-FTO lentiviral overexpression plasmid for gene overexpression, or with Si-FTO and NC-FTO (negative control siRNA) for gene knockdown. For differentiation experiments, rabbit preadipocytes were transfected with the respective constructs after reaching >80% confluence. Transfection was performed using Lipofectamine 3000 (Invitrogen, Carlsbad, CA, USA) for 6 h followed by replacement with differentiation induction medium (Table 1).

### 2.4. Oil Red O Staining Protocol

Preadipocytes cultured in 35 mm dishes (NEST Biotechnology, Wuxi, China) were washed twice with PBS and fixed with 10% formaldehyde for 30 min. The Oil Red O stock solution (NJBI, Nanjing, China) was diluted 3:2 with DEPC water, filtered, and applied as working solution for 25 min staining. After PBS washing, lipid droplet formation was documented under an inverted microscope (Olympus, Tokyo, Japan). For quantification, 2 mL isopropanol was added to extract stained lipids with the eluate transferred to 96-well plates for absorbance measurement at 510 nm using a the Thermo Fisher Scientific Varioskan LUX microplate (Waltham, MA, USA) reader. Statistical analysis used GraphPad Prism 9 (v9.0.1; GraphPad Software, La Jolla, CA, USA).

### 2.5. CCK-8 Viability Assay

Fourth-passage rabbit preadipocytes were plated in 96-well plates (6 replicates per group, 100 µL medium/well) and transfected at ~70% confluence. Following the manufacturer’s protocol, 10 µL CCK-8 reagent was added to each well at 0, 24, 48, and 72 h post-transfection, followed by 2 h incubation at 37 °C with 5% CO_2_. A450 was quantified using a Varioskan LUX microplate reader (Thermo Fisher Scientific, Waltham, MA, USA); data was processed with Prism 9.

### 2.6. Gene Expression Profiling: RNA to qPCR

RNAiso reagent (Takara, Beijing, China) was used to isolate total RNA from biological samples according to supplier’s protocol. RNA purity was examined through measuring A260/A280 and A260/230 ratios using a NanoDrop 2000 spectrophotometer (Thermo Fisher Scientific, Waltham, MA, USA). cDNA synthesis was performed with the PrimeScript RT Reagent Kit (Takara) according to the manufacturer’s instructions, and the resulting cDNA was stored at −20 °C until use. For qPCR analysis, reactions were carried out in 10 µL volumes using SYBR Green-based qPCR using Best Enzyme mix (Shanghai, China) on CFX96 RT-PCR system (Bio-Rad, Hercules, CA, USA) with GAPDH serving as the housekeeping gene. Primer sequences are listed in Table 2, and relative gene expression levels were calculated using the 2^−ΔΔCt^ method.

### 2.7. Western Blot Analysis

Total proteins were isolated with a Solarbio extraction kit (Beijing, China) per manufacturer’s protocol. Concentrations were quantified by Bradford assay (Novoprotein, Shanghai, China). Equal protein aliquots were electrophoresed on 10% SDS-PAGE and trans-blotted to PVDF membranes. After blocking in 5% non-fat milk, the membranes were incubated with primary antibodies at 4 °C overnight, followed by incubation with HRP-conjugated secondary antibodies (goat anti-rabbit IgG, Zen Bioscience, Chengdu, China) for 2 h at RT. Membranes were TBST-washed (3×) prior to ECL detection on Touch Imager Pro (e-BLOT Life Science, Shanghai, China). Primary antibodies: PCNA/FTO from Bioworld Technology (Chengdu) and PPARγ/β-actin from Abclonal (Wuhan, China). Table 3 lists the antibody dilution concentrations used.

### 2.8. RNA Sequencing and Bioinformatics Analysis

RNA samples underwent quality control, with quantification by Qubit 3.0 Fluorometer confirming concentrations ≥ 1 ng/μL Simultaneously, RNA integrity was assessed using an Agilent 2100 Bioanalyzer (Thermo Fisher Scientific, Waltham, MA, USA) to obtain RNA Integrity Number (RIN) scores, with ideal values ranging between 7 and 10 to ensure reliability of downstream experiments. Library insert size was evaluated using the QSEP400 High-Throughput Analysis System (Bioptic, New Taipei City, Taiwan, China). When insert size met predefined criteria, RT-qPCR was employed to precisely quantify effective library concentration (effective concentration > 2 nM). Following a successful library quality inspection, individual libraries were pooled according to their effective concentrations and required off-target data volume. Pooled libraries were then sequenced on an Illumina platform (San Diego, CA, USA). During sequencing: DNA molecular anchors and fluorescent probes were polymerized onto DNA nanoballs through combinatorial probe synthesis technology. Optical signals were captured via a high-resolution imaging system and subsequently digitized to generate high-quality, high-accuracy sequence data. DESeq2 identified differentially expressed genes at * *p* ≤ 0.05.

### 2.9. Statistical Analysis

Data expressed as mean ± SEM. GraphPad Prism 9 was used for additional analyses. Group differences were assessed by Student’s *t*-test; multiple comparisons by one-way ANOVA (* *p* < 0.05 and ** *p* < 0.01).

## 3. Results

### 3.1. Effects of Live Weight Before Slaughter-on-Slaughter Traits and Meat Quality Traits in Meat Rabbits

**Figure 1:**
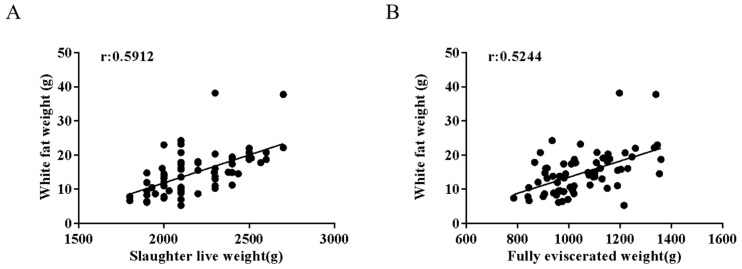
Effects of live weight before slaughter-on-slaughter traits and meat quality in meat rabbits. (**A**) Correlation between live weight before slaughter and fat weight (*n* = 70). (**B**) Correlation between fully eviscerated yield and fat weight (*n* = 70). Correlation coefficient r: It is used to measure the degree of correlation between two variables. The value range of r is [−1, 1]. A value range of [0.6–1.0] for r represents a strong positive correlation, [0.4–0.6] represents a moderate positive correlation, [0.0–0.4] represents a weak positive correlation, and the same logic applies to negative correlations.

To investigate the effects of live weight before slaughter-on-slaughter performance and the meat quality of Ira rabbits, we conducted a study where rabbits were raised to 70 days of age and divided into two groups based on market weight (2.25 kg). We randomly selected 35 meat rabbits each from the low-body-weight group (below 2.25 kg) and the high-body-weight group (above 2.25 kg) for sampling. We measured slaughter traits including live weight, eviscerated yield (full and partial), abdominal fat weight, and perirenal fat weight. The results demonstrated significant positive correlations between live weight and fat deposition (Figure 1A), as well as between eviscerated yield and fat weight (Figure 1B). The high-body-weight group showed markedly higher abdominal and perirenal fat weights (*p* < 0.01), indicating enhanced fat deposition, along with improved slaughter yield and meat production (Table 4). Further analysis of meat quality traits 24 h post-slaughter revealed that the high-body-weight group exhibited significantly reduced drip loss (*p* < 0.05), suggesting better water-holding capacity due to increased fat content. Cooking yield was also higher (*p* < 0.05), attributable to improved fat and moisture retention. Additionally, the high-body-weight group displayed more rapid pH decline in the longissimus dorsi muscle, likely resulting from greater glycogen reserves and subsequent lactic acid accumulation (Table 5). These findings collectively indicate that higher pre-slaughter live weight in rabbits promotes fat deposition, improves slaughter performance, and enhances key meat quality characteristics.

### 3.2. Serum Adiponectin, Leptin, and Total Cholesterol Levels in Meat Rabbits

**Figure 2:**
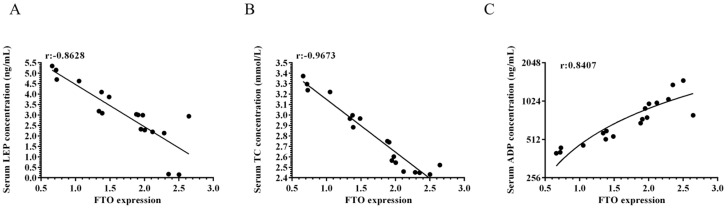
Serum adiponectin, leptin, and total cholesterol levels in meat rabbits. (**A**) Correlation between FTO expression and serum leptin (LEP) levels (*n* = 18). (**B**) Correlation between FTO expression and serum total cholesterol (TC) levels (*n* = 18). (**C**) Correlation between FTO expression and serum adiponectin (ADP) levels (*n* = 18). Correlation coefficient r: It is used to measure the degree of correlation between two variables. The value range of r is [−1, 1]. A value range of [0.6–1.0] for r represents a strong positive correlation, [0.4–0.6] represents a moderate positive correlation, [0.0–0.4] represents a weak positive correlation, and the same logic applies to negative correlations.

In this study, we measured serum levels of adiponectin (ADP), leptin (LEP), and total cholesterol (TC) in meat rabbits at 0, 35, and 70 days of age. Our findings revealed that FTO expression in adipose tissue showed a negative correlation with serum LEP (r = −0.8628) and TC levels (r = −0.9673), while demonstrating a positive correlation with serum ADP levels (r = 0.8407) (Figure 2A–C). These results suggest that FTO may play a regulatory role in lipid metabolism and adipokine secretion during rabbit growth and development.

### 3.3. FTO Is Associated with Adipose Tissue Development

**Figure 3:**
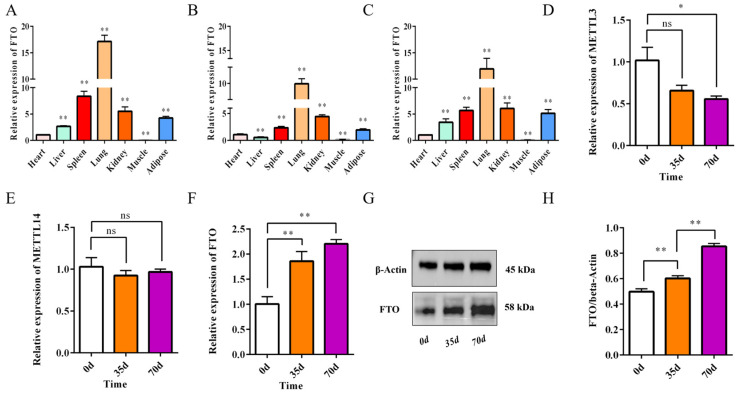
Association between FTO and adipose tissue development. (**A**–**C**) Tissue-specific expression profiles of FTO at (**A**) 0, (**B**) 35, and (**C**) 70 days of age (*n* = 6). (**D**,**E**) Relative mRNA expression of (**D**) METTL3 and (**E**) METTL14 in perirenal adipose tissue across developmental stages (no significant changes) (*n* = 6). (**F**) Age-dependent increase in FTO mRNA expression in adipose tissue (** *p* < 0.01) (*n* = 6). (**G**,**H**) Western blot analysis confirming progressive upregulation of FTO protein expression during development (*n* = 3). Data are presented as mean ± SEM. * *p* < 0.05 and ** *p* < 0.01. ns (no significance).

In this study, we examined FTO expression in seven tissues (including heart, liver, and lung) of Ira rabbits at 0, 35, and 70 days of age using qPCR (Figure 3A–C). The results consistently demonstrated that FTO was most highly expressed in the lung and least expressed in the leg muscle, with relatively high expression in adipose tissue, showing a significant difference compared to the heart (*p* < 0.05). Additionally, we collected perirenal adipose tissue from Ira rabbits at 0, 35, and 70 days of age. qPCR analysis revealed no significant changes in the expression of METTL3 and METTL14 in adipose tissue across these developmental stages (Figure 3D,E). In contrast, FTO expression significantly increased with age (*p* < 0.01), a finding further confirmed by the Western blot (WB) analysis (Figure 3F–H). Together, these results indicate that FTO is closely associated with adipose tissue development in meat rabbits.

### 3.4. Establishment of a Preadipocyte Differentiation Model in Meat Rabbits

**Figure 4:**
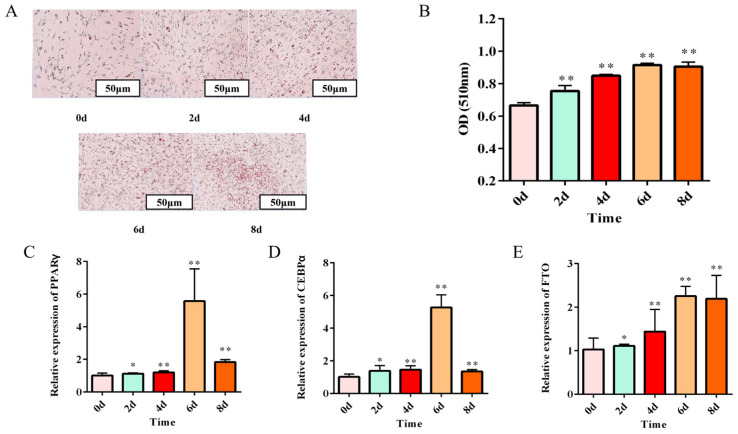
Establishment of a preadipocyte differentiation model in meat rabbits. (**A**) Oil Red O: lipid droplets (d0, 2, 4, 6, and 8) (**B**) Oil Red O OD quantitation (OD510) (*n* = 3). (**C**) Relative mRNA expression of PPARγ during differentiation (*n* = 6). (**D**) Relative mRNA expression of C/EBPα during differentiation (*n* = 6). (**E**) Relative mRNA expression of FTO during differentiation (*n* = 6). Data are presented as mean ± SEM. * *p* < 0.05 and ** *p* < 0.01 versus day 0 control.

We successfully established an in vitro differentiation model of rabbit preadipocytes by inducing differentiation at 80% confluence. Oil Red O staining demonstrated progressive lipid droplet accumulation and enlargement during differentiation (Figure 4A), which was quantitatively confirmed by spectrophotometric analysis showing increasing optical density at 510 nm (Figure 4B). Molecular characterization revealed time-dependent upregulation of key adipogenic transcription factors, PPARγ and C/EBPα, peaking at day 6 and maintaining significantly elevated levels at day 8 compared to baseline (day 0) (Figure 4C,D). Notably, qPCR analysis identified a parallel increase in FTO expression throughout the differentiation process (Figure 4E). These findings collectively validate our differentiation model through morphological, biochemical, and molecular evidence, while suggesting FTO’s potential involvement in adipocyte differentiation. (Data presented as mean ± SEM; * *p* < 0.05 and ** *p* < 0.01 vs. day 0).

### 3.5. Analysis of Differentially Expressed Genes

To further elucidate the functional mechanisms of FTO, we performed transcriptome sequencing analysis comparing pcDNA3.1-FTO transfected cells with pcDNA3.3.1 vector controls in rabbit preadipocytes. Six high-quality cDNA libraries were successfully constructed, yielding 237,010,838 clean reads after rigorous quality filtering using Cutadapt. The sequencing data exhibited excellent quality metrics, with Q20 and Q30 scores ranging 99.64–99.67% and 96.53–96.90%, respectively, indicating superior base-calling accuracy. The GC content remained stable across samples (53–53.5%, mean = 53.25%), and an average of 86.20% reads were successfully mapped to the reference genome, confirming the reliability of our sequencing data for subsequent differential gene expression analysis. These comprehensive quality control results demonstrate that our transcriptome datasets meet all stringent requirements for in-depth bioinformatics analysis of FTO-mediated regulatory networks in adipogenesis. (Note: Q20/Q30 represents the percentage of bases with quality scores ≥ 20/30, respectively).

**Figure 5:**
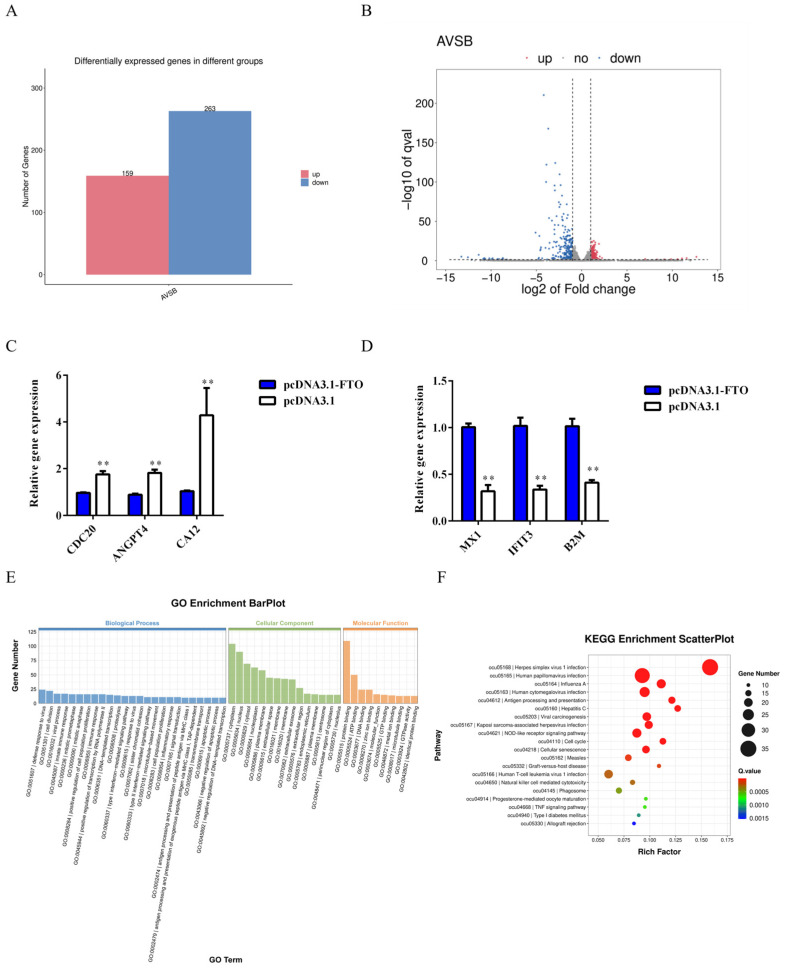
Analysis of differentially expressed genes (DEGs). (**A**) Distribution of 422 identified DEGs (159 upregulated and 263 downregulated). (**B**) DEG volcano plot. *X*-axis: log_2_FC; *Y*-axis: −log_10_(p). (**C**,**D**) qRT-PCR validation of (**C**) upregulated and (**D**) downregulated DEGs (*n* = 6 randomly selected genes) (*n* = 6). (**E**) GO enrichment analysis of DEGs across 1923 terms (1342 biological processes, 230 cellular components, and 351 molecular functions). (**F**) KEGG pathway enrichment showing top significantly enriched pathways. Data presented as mean ± SEM. ** *p* < 0.01.

Subsequent analysis using DESeq2 identified 422 known differentially expressed genes (DEGs) from the sequencing libraries, including 159 upregulated and 263 downregulated genes (Figure 5A). A volcano plot was constructed to visualize these DEGs based on log2(fold change) and −log10(*p*-value) values (Figure 5B). qRT-PCR validation of six randomly selected DEGs confirmed consistent expression trends with the sequencing results (Figure 5C,D). Functional enrichment analysis revealed significant GO term enrichment across 1923 categories, comprising 1342 biological processes (BP), 230 cellular components (CC), and 351 molecular functions (MF) (Figure 5E). The key biological processes included antigen processing and presentation, virus detection, and regulation of white adipocyte proliferation. KEGG pathway analysis identified enrichment in 227 pathways, with natural killer cell-mediated cytotoxicity, type I diabetes mellitus, and TNF signaling pathway showing particularly significant enrichment (Figure 5F).

### 3.6. Effect of FTO on the Proliferation of Rabbit Preadipocytes

**Figure 6:**
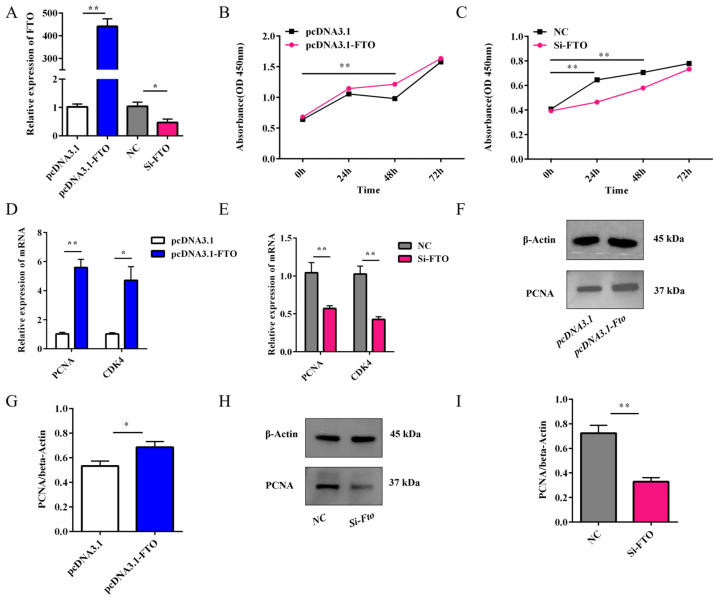
Effects of FTO on the proliferation of rabbit preadipocytes. (**A**) Transfection efficiency of FTO overexpression (OE) and knockdown (KD) (*n* = 6). (**B**) CCK-8 assay results at 0, 24, 48, and 72 h post-OE (*n* = 6). (**C**) Viability (CCK-8) post-KD. Time points: 0, 24, 48, and 72 h (*n* = 6). (**D**) PCNA expression and CDK4 expression after OE (*n* = 6). (**E**) Relative mRNA expression of PCNA and CDK4 after KD (*n* = 6). (**F**,**G**) PCNA protein expression levels after OE (*n* = 3). (**H**,**I**) PCNA protein expression levels after KD (*n* = 3). Data are presented as mean ± SEM. * *p* < 0.05 and ** *p* < 0.01 versus respective controls.

To investigate the proliferative role of FTO in rabbit preadipocytes, we performed gain- and loss-of-function experiments by transfecting cells with either pcDNA3.1-FTO (for overexpression) or Si-FTO (for knockdown), along with their respective controls (pcDNA3.1 and NC). Successful modulation of FTO expression was confirmed (Figure 6A). CCK-8 assays conducted 72 h post-transfection demonstrated that FTO overexpression significantly enhanced cell proliferation (*p* < 0.05), while FTO knockdown suppressed proliferative activity compared to controls (Figure 6B,C). Molecular analysis revealed that FTO overexpression upregulated the expression of proliferation marker genes PCNA and CDK4 at both mRNA (*p* < 0.05, Figure 6D,E) and protein levels (Figure 6F–I), whereas FTO knockdown produced the opposite effects. These results collectively demonstrate that FTO exerts a positive regulatory effect on preadipocyte proliferation, likely through modulating the expression of key cell cycle regulators PCNA and CDK4.

### 3.7. Effect of FTO on the Differentiation of Rabbit Preadipocytes

**Figure 7:**
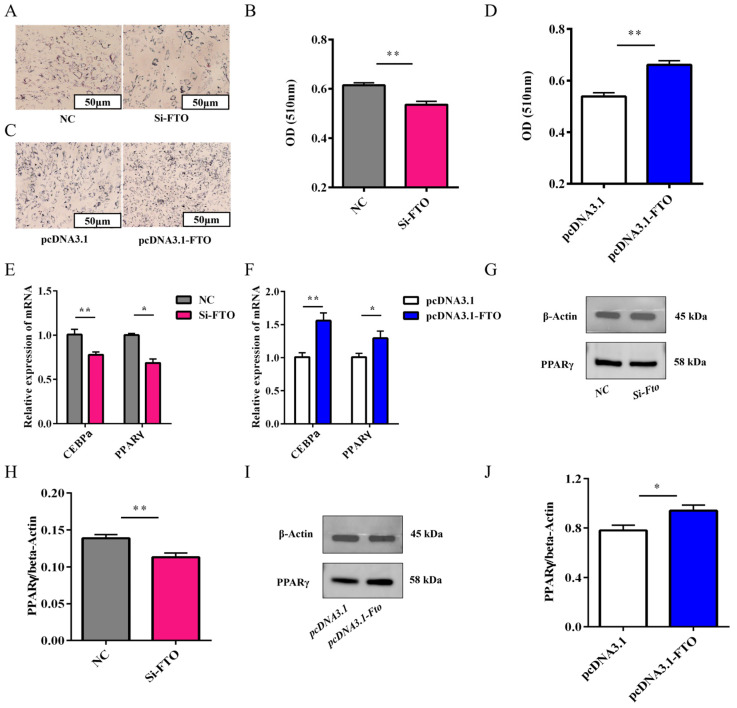
Effects of FTO on rabbit preadipocyte differentiation. (**A**) Lipid staining (Oil Red O) droplets after FTO knockdown. (**B**) Quantitative analysis of Oil Red O staining (OD values) after knockdown (*n* = 3). (**C**) Oil Red O staining after FTO overexpression. (**D**) Quantitative analysis of Oil Red O staining (OD values) after overexpression (*n* = 3). (**E**) Relative mRNA expression of C/EBPα and PPARγ after FTO knockdown. (**F**) Relative mRNA expression of C/EBPα and PPARγ after FTO overexpression (*n* = 6). (**G**,**H**) PPARγ protein expression levels after FTO knockdown (*n* = 3). (**I**,**J**) PPARγ protein expression levels after FTO overexpression (*n* = 3). Data are presented as mean ± SEM. * *p* < 0.05 and ** *p* < 0.01 versus respective controls.

Defining FTO’s role in adipogenesis, fourth-generation preadipocytes were cultured in cell culture dishes and transfected before differentiation induction. Oil Red O staining and microscopic observation showed that FTO knockdown resulted in fewer lipid droplets in cells and significantly reduced OD values (Figure 7A,B). In contrast, FTO overexpression promoted lipid droplet formation in adipocytes and significantly increased OD values (Figure 7C,D). qPCR results demonstrated that compared with the control group, FTO knockdown significantly decreased the expression of adipogenic marker genes C/EBPα and PPARγ (Figure 7E), while overexpression significantly increased their expression (Figure 7F). A Western blot analysis further verified these findings at the protein level (Figure 7G–J). Therefore, FTO overexpression promotes rabbit preadipocyte differentiation, whereas FTO knockdown inhibits this process.

## 4. Discussion

Obesity is defined by the World Health Organization as the excessive accumulation of white adipose tissue. Research indicates that white adipose tissue deposition in livestock is closely associated with organismal development [16]. During early growth stages, an animal’s energy is preferentially allocated to lean tissue synthesis, while adipose deposition accelerates in later growth phases [17]. Our in vivo observations revealed a significant increase in FTO expression during adipose tissue development, which aligns with the results from in vitro experiments showing that FTO overexpression promotes adipocyte differentiation. This suggests that upregulation of FTO expression may be an important molecular mechanism underlying the rapid adipose tissue deposition in meat rabbits during the later stages of growth. Adipose tissue deposition plays a crucial role in meat quality formation, as its differentiation efficiency and patterns directly determine intramuscular fat content and fatty acid composition, thereby influencing meat tenderness, flavor, and juiciness [18,19,20,21,22]. Our investigation into the effects of pre-slaughter live weight on slaughter traits and meat quality in rabbits revealed a positive correlation between live weight and fat mass, as well as between eviscerated carcass weight and fat mass. Furthermore, higher-weight rabbits exhibited more substantial white adipose deposition, significantly improved slaughter yield and meat production traits, reduced drip loss, and increased cooking yield—all contributing to enhanced meat quality. Therefore, for inherently lean rabbit meat, moderate fat accumulation can improve flavor and tenderness, thereby boosting its market competitiveness.

Studies on the variation in FTO protein expression in porcine cells with age, diet, and metabolism revealed that FTO expression in the pancreas positively correlates with energy intake, whereas in muscle tissue, it is age-dependent. Notably, FTO protein levels in the cerebellum and kidneys were significantly higher in growth-retarded pigs compared to those with normal birth weight [23]. Expression profiling of FTO across different tissues in meat rabbits demonstrated elevated expression in adipose tissue while pulmonary expression was markedly higher than in all other examined tissues suggesting a potential regulatory role in lung development. Further research has established that FTO plays a critical role in modulating the growth and invasiveness of lung cancer cells by influencing mRNA stability and translation efficiency [24]. In the present study, FTO expression increased significantly during adipocyte differentiation leading us to hypothesize that elevated m^6^A modification in adipocytes promotes FTO expression.

Adipose tissue development is an essential process in animal growth and a key research focus in modern animal science [25]. Adipocyte proliferation serves as the foundation for adipose tissue development and injury repair while abnormal proliferation in obesity leads to adipose tissue hyperplasia and increased risk of metabolic disorders [26]. Studies demonstrate that the ATM COX-2/PGE2/EP4 axis plays a critical role in mitigating adipose tissue dysfunction in mice. When fed a high-fat diet (HFD), adipose tissue macrophages (ATMs) upregulate COX-2 expression, resulting in pathological adipose tissue angiogenesis [27]. Adipocyte differentiation refers to the process whereby preadipocytes mature into functional adipocytes under specific signaling cues to maintain metabolic homeostasis [28]. Impaired differentiation can cause ectopic lipid deposition, leading to insulin resistance and metabolic syndrome [29].

Our study demonstrates that FTO overexpression at the cellular level promotes the proliferation and differentiation of rabbit preadipocytes whereas FTO knockdown inhibits these processes. These findings align with previous reports on FTO’s regulatory role in porcine intramuscular preadipocytes [30], suggesting functional conservation of FTO-mediated adipocyte regulation across mammalian species. In porcine preadipocytes, FTO promotes cell differentiation and lipid droplet accumulation by demethylating mRNA of genes associated with the JAK2-STAT3-C/EBPβ signaling pathway [12]. For example, FTO overexpression significantly increases triglyceride (TG) content and upregulates the expression of differentiation marker genes such as PPARγ and C/EBPα. FTO is also involved in regulating the autophagy pathway, promoting adipocyte differentiation by stabilizing the mRNA of ATG5 and ATG7 [9]. Furthermore, in avian (chicken) preadipocytes, FTO promotes differentiation by demethylating and regulating the mRNA stability of CTNNB1 (β-catenin), thereby inhibiting the Wnt/β-catenin pathway [31]. FTO promotes rabbit preadipocyte differentiation by reducing the m^6^A modification of the ADRB1 gene (an anti-differentiation factor), thereby enhancing its mRNA stability [32].

This study, from the macroscopic production performance and in vivo experiments to the microscopic molecular mechanisms (cellular experiments) is the first to reveal the regulatory role of FTO in the proliferation and differentiation of adipocytes in meat rabbits, providing a novel model for livestock breeding and obesity research. Future studies should focus on the following: (1) Identifying downstream target genes through sequencing to elucidate m^6^A modification’s role in adipogenesis, and (2) performing phenotypic analysis of FTO-knockout rabbits for in vivo validation.

## 5. Conclusions

The results demonstrate that FTO overexpression promotes the proliferation and differentiation of rabbit preadipocytes, while FTO interference inhibits these processes. These findings not only deepen our understanding of adipose metabolism mechanisms, but also provide novel insights for agricultural breeding optimization, livestock meat quality improvement, and therapeutic strategies for obesity and related metabolic disorders.

## Figures and Tables

**Table 1:** FTO-targeting siRNA sequences.

Name	Primer Sequences
*Si-FTO-F*	GUGAAAGGUUCUACUAUAA
*Si-FTO-R*	UUAUAGUAGAACCUUUCAC

**Table 2:** Primer sequences.

Name	Primer Sequence
*GAPDH-F*	*CTGACCTGCCGCCTGGAGAAAG*
*GAPDH-R*	*CCTGTTGCTGTAGCCAAATTCGTTG*
*FTO-F*	CGCGAGCGTGAAGCTAAGAAA
*FTO-R*	TTATGGAGCTCCTCGGACAC
*PPARγ-F*	CGCTGATGCACTGCCTATGA
*PPARγ-R*	AGAGGTCCACAGAGCTGATTCC
*C/EBPα-F*	*CAAGAACAGCAACGAGTACCG*
*C/EBPα-R*	*GTCACTGGTCAACTCCAGCAC*
*CDK4-F*	*AGTTTCTAAGCGGCCTGGAT*
*CDK4-R*	*AACTTCAGGAGCTCGGTACC*
*PCNA-F*	*TTGCACGTATATGCCGAGACC*
*PCNA-R*	*GGTGAACAGGCTCATTCATCTCT*
*METTL3-F*	*GTCCCCAACCTTCCGTAGTG*
*METTL3-R*	*TGTGCTAGACTTGGAGCCAC*
*METTL14-F*	*GATAGCCGCTTGCAGGAGAT*
*METTL14-R*	*TGCTGTTTAGCACAGCACCT*

**Table 3:** Antibody dilution concentration.

Antibody	Company	Accession Number	Dilution Ratio
anti-PCNA	Zenbio (Chengdu, China)	R25293	1:3000
anti-PPARγ	Nature Biosciences (Shanghai, China)	#51311	1:1000
anti-β-Actin	GenuIN (Hefei, China)	#2885	1:5000
anti-FTO	Proteintech (WuHan, China)	27226-1-AP	1:1500

**Table 4:** Effect of live weight before slaughter-on-slaughter traits of meat rabbits.

Slaughtering Traits	Slaughter Liveweight (g)	Fully Eviscerated Weight (g)	Eviscerated Yield (%)	Semi-Eviscerated Yield (%)	Abdominal and Renal Fat Weights (g)
Low-BW Group (*n* = 35)	2007.00 ± 14.26	944.80 ± 8.87	47.05 ± 0.13	51.45 ± 0.13	11.80 ± 0.57
High-BW Group (*n* = 35)	2421.60 ± 20.65 **	1156.20 ± 15.88 *	47.68 ± 0.23 *	52.23 ± 0.26 *	18.42 ± 0.93 **

Note: Data are expressed as mean ± SEM; * indicates *p* < 0.05 and ** indicates *p* < 0.01.

**Table 5:** Effect of live weight before slaughter on meat quality traits of rabbits.

Meat Traits	Index Drip Loss (%)	Cooking Rate (%)	Leg Muscle pH	Abdominal and Renal Fat Weights (g)
Low-BW Group (*n* = 35)	7.17 ± 0.12	64.50 ± 0.14	5.83 ± 0.02	11.80 ± 0.57
High-BW Group (*n* = 35)	6.55 ± 0.09 **	64.97 ± 0.12 *	5.62 ± 0.02 **	18.42 ± 0.93 **

Note: Data are expressed as mean ± SEM; * indicates *p* < 0.05 and ** indicates *p* < 0.01.

## Data Availability

The original Western blot images presented in this study are available in Supplementary Materials.

## Supplementary Materials

The following supporting information can be downloaded at: https://www.mdpi.com/article/10.3390/ani15131909/s1, File S1. Original Western blot images.

## Abbreviations

CDK4	Cyclin-Dependent Kinase 4
C/EBPα	CCAAT/Enhancer-Binding Protein Alpha
PCNA	Proliferating Cell Nuclear Antigen
PPARγ	Peroxisome Proliferator-Activated Receptor Gamma
β-actin	Beta-Actin
