# Supplementary figures and images for: The Effects of FTO on the Proliferation and Differentiation of Rabbit Preadipocytes
Source: Animals (Basel). 2025 Jun 28;15(13):1909. doi: 10.3390/ani15131909 (PMC12249043; doi:10.3390/ani15131909)

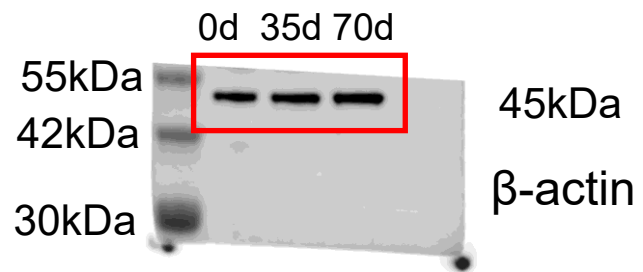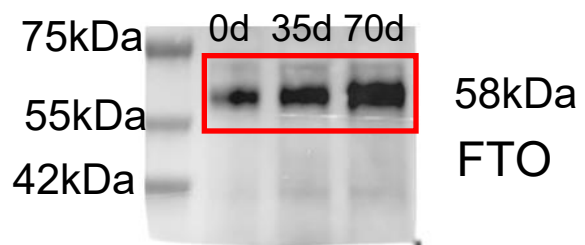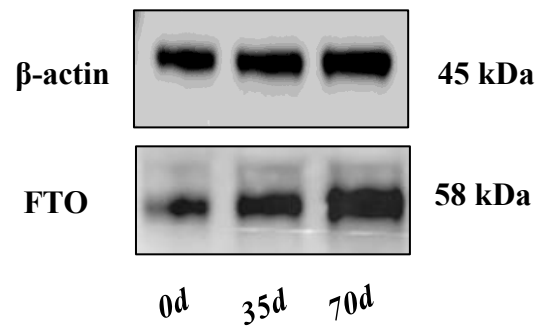

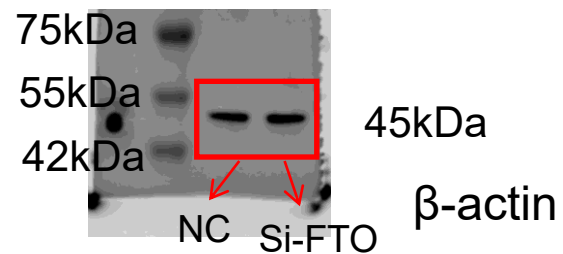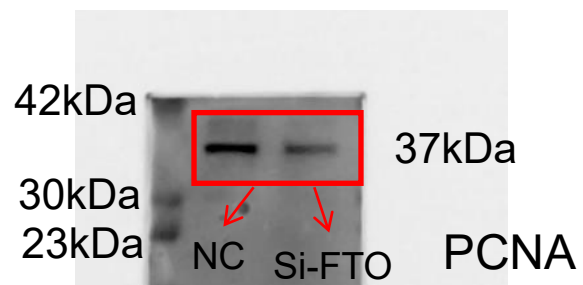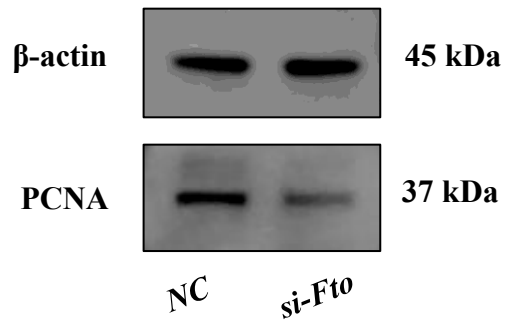

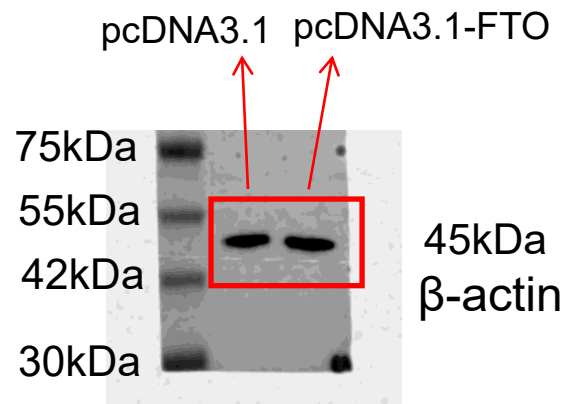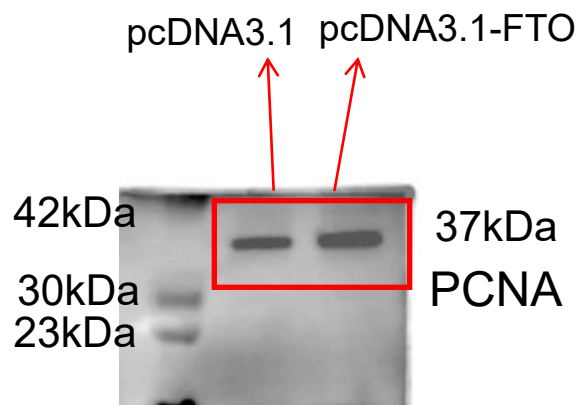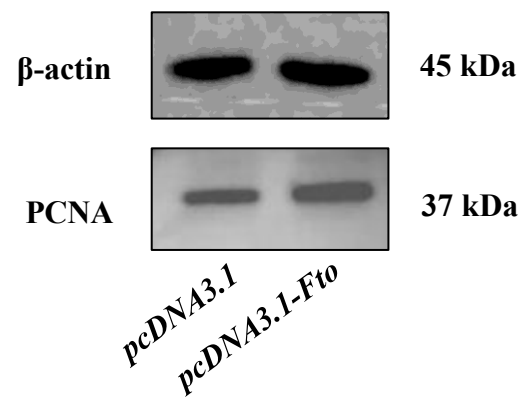

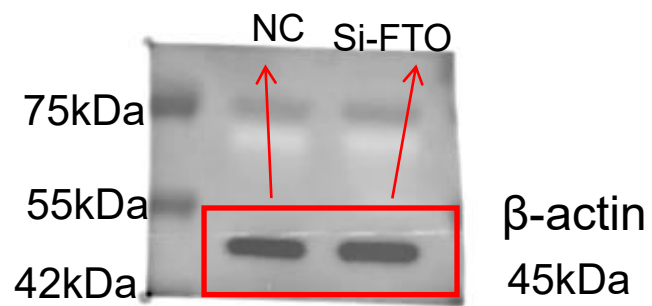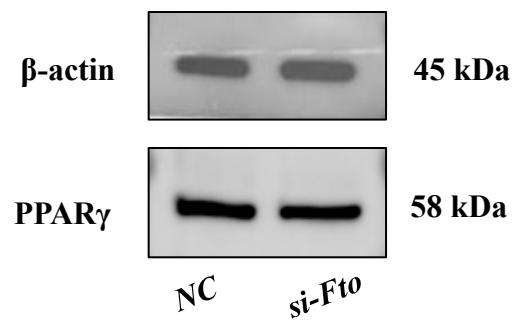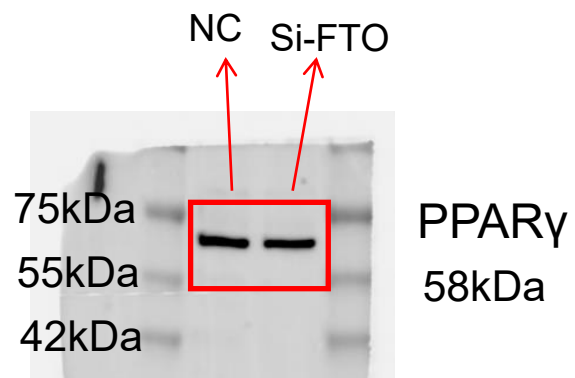

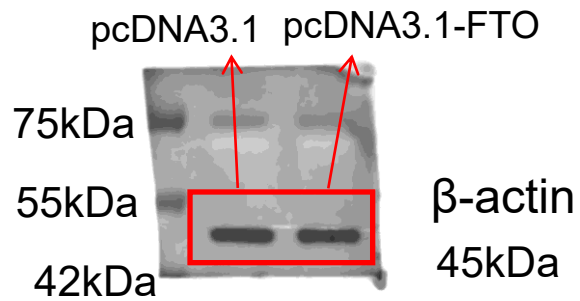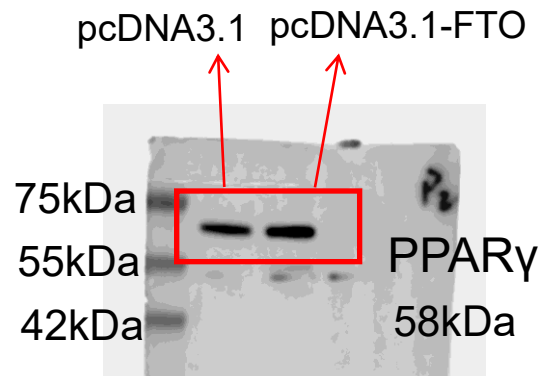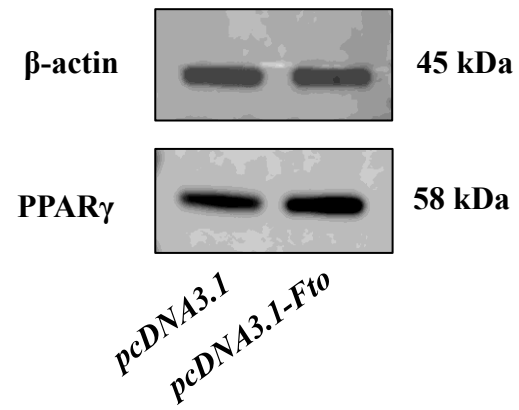

Supplement: Supplementary file 1 [file animals-15-01909-s001.zip › animals-3724655-supplementary.pdf]
